# Coronavirus and Pregnancy: The Challenges of the 21^st^ Century: A Review

**DOI:** 10.3389/fmicb.2022.923546

**Published:** 2022-07-13

**Authors:** Sumaiya Adam, Carmen Pheiffer, Stephanie Dias, Tsakane Hlongwane, Valerie Vannevel, Priya Soma-Pillay, Fareed Abdullah

**Affiliations:** ^1^Department of Obstetrics and Gynecology, Faculty of Health Sciences, University of Pretoria, Pretoria, South Africa; ^2^Research Centre for Maternal, Fetal, Newborn and Child Health Care Strategies, University of Pretoria, Pretoria, South Africa; ^3^Biomedical Research and Innovation Platform, South African Medical Research Council, Cape Town, South Africa; ^4^Centre for Cardio-metabolic Research in Africa (CARMA), Division of Medical Physiology, Faculty of Medicine and Health Sciences, Stellenbosch University, Cape Town, South Africa; ^5^Office of AIDS and TB Research, South African Medical Research Council, Pretoria, South Africa; ^6^Division of Infectious Diseases, Department of Internal Medicine, Faculty of Health Sciences, University of Pretoria, Pretoria, South Africa

**Keywords:** SARS-CoV-2, pregnancy, coronavirus, MERS, health systems

## Abstract

Despite many advances in medicine we are still faced with emerging pathogens. Pregnant women have been disproportionately affected by previous coronavirus outbreaks. The COVID-19 pandemic has not affected pregnant women as greatly as SARS-CoV and MERS, but has posed other challenges such as the need for quarantine and isolation, limited access to antenatal care, use of personal protective equipment (PPE), vaccine hesitancy and inequities in vaccine access and therapeutics between rich countries and the global south. This review will describe the impact of the significant coronaviruses on pregnancy, with special focus on the challenges being encountered by the SARS-CoV-2 global pandemic.

## Introduction

As we celebrate advances in obstetric care, improved maternal and perinatal outcomes, and a move toward personalized medicine, challenges such as human immunodeficiency virus/acquired immunodeficiency syndrome (HIV/AIDS) and emerging pathogens, still plague us (Abdelrahman et al., 2020; Di Mascio et al., 2020; Zhu et al., 2020; Matar et al., 2021). COVID-19, which is caused by the SARS-CoV-2 virus, has been associated with poor outcomes for mother (increased mortality and admission to critical care) and newborn [preterm labor and admission to the neonatal intensive care unit (NICU)] (Kim et al., 2015; Abdelrahman et al., 2020; Czeresnia et al., 2020; Di Mascio et al., 2020; Zhu et al., 2020; Matar et al., 2021). The SARS-CoV-2 pandemic and its manifestation in the global cohort of pregnant women reminds us of our vulnerabilities, gives us pause to reflect on the other novel infections that have challenged human health over the last 20 years; and to contemplate how we will react to future challenges to global health. This review will describe the impact of the significant coronaviruses on pregnancy, with special focus on the challenges being encountered by the SARS-CoV-2 global pandemic.

## The Coronaviruses

Coronaviruses are a group of pathogens with an animal reservoir that cause contagious, and sometimes fatal respiratory diseases in humans (Abdelrahman et al., 2020; Zhu et al., 2020). The coronaviruses were amongst the first major infectious pathogens to emerge in the 21st century. Coronaviruses can be spread *via* air in small airborne droplets of saliva, or indirectly spread *via* contaminated surfaces touched by an infected individual (Di Mascio et al., 2020; Zhu et al., 2020). The first coronavirus was identified in 2003. Since then, seven human coronaviruses have been identified, namely, *Human coronavirus HKU1* (*HCoV-HKU1*), *human coronavirus OC43* (HCoV-OC43), *human coronavirus 229E* (HCoV-229E), *human coronavirus NL63* (HCoV-NL63), Middle East respiratory syndrome coronavirus (MERS-CoV), severe acute *respiratory syndrome coronavirus 1* (SARS-CoV-1 or SARS-CoV), and SARS-CoV-2. The last three viruses have resulted in severe illness in humans and warrant further discussion (Abdelrahman et al., 2020; Czeresnia et al., 2020; Di Mascio et al., 2020; Zhu et al., 2020).

Whilst there are many similarities between SARS-CoV-1, MERS, and SARS-CoV-2, there are also significant differences, which are summarized in Table 1.

**TABLE 1:** Comparison of SARS-CoV-1, MERS, and SARS-CoV-2.

	SARS-CoV-1	MERS	SARS-CoV-2
First emergence	November, 2002	April, 2012	December, 2019
Regions affected			
Emerged	China	Jordan	China
Spread to	China, Vietnam, Hong Kong, Singapore, Taiwan, Canada	Saudi Arabia, Jordan, Yemen. Few cases in United States	Worldwide pandemic
Natural host	Bats	Bats	?Bats, ?pangolins
Intermediate animal host	Palm civet, ?bats	Camel, ?bats	?Bats, ?pangolins (controversial)
Common symptoms	Fever, tiredness, dry cough, myalgia, nasal congestion, sore throat, diarrhea, lymphopenia	Cough, shortness of breath, headache, sore throat, fever, loss of appetite, diarrhea, nausea, and vomiting	Fever, shortness of breath, sore throat, cough, tiredness, anosmia, dysgeusia, diarrhea. Leukocytosis (?pregnancy related) (Magee et al., 2021)
Genomic structure	Single-stranded RNA (ssRNA)	Single-stranded RNA (ssRNA) (Kim et al., 2015)	Positive single-stranded RNA virus
	20% less ACE2- binding affinity than SARS-CoV-2 (Zhu et al., 2020)		Shares 80% sequence identity with SARS- CoV-1 and 50% similarity with MERS (Naqvi et al., 2020)
Variants of concern	TOR2, Urbani,	MH013216,	Alpha, Beta,
	CUHK-W1, CUHK-	MN120513,	Gamma,
	Su10, HKU-39849,	MN120514,	Delta, Epsilon, Eta,
	SIN2500, SIN2677,	MH306207,	Iota, Kappa, 1.617.3,
	SIN2679, SIN2748,	MH359139,	Mu, Zeta
	SIN2774, TW1, BJ01,	MH371127,	Omicron
	BJ02, BJ03, BJ04, GZ01	MH432120, MH454272	
Cases	8,098 reported cases	2,578 confirmed cases	397 million cases to date
Reinfection	No reported cases	No reported cases	+++
Case fatality rate	9.6%	34.3%	4.4%

SARS-CoV-1 causes a respiratory illness which was responsible for the 2002–2004 SARS outbreak (Abdelrahman et al., 2020; Di Mascio et al., 2020; Zhu et al., 2020; Matar et al., 2021). It is an enveloped, positive-sense, single-stranded RNA virus which infects the epithelial cells within the lungs. The virus enters the host cell by binding to angiotensin-converting enzyme 2 (ACE2) receptor. It infects humans, bats, and palm civets. SARS-CoV-1 caused a severe illness characterized by systemic symptoms of myalgia, headache, and fever, followed by the onset of respiratory symptoms such as mainly cough, dyspnea, and pneumonia, and lymphopenia within 2 weeks. In the SARS-CoV-1 outbreak of 2003, about 9% of patients with confirmed infection died (Abdelrahman et al., 2020; Czeresnia et al., 2020).

MERS-CoV is a viral respiratory disease that was first identified in Saudi Arabia in 2012 (Kim et al., 2015; Abdelrahman et al., 2020; Di Mascio et al., 2020; Zhu et al., 2020). Infection with MERS-CoV ranged from asymptomatic infection to typical symptoms such as fever, cough, shortness of breath, and occasional diarrhea. Pneumonia was common, but not always present. Dromedary camels, which are a major reservoir host for MERS-CoV, are implicated in the transmission of MERS-CoV to humans, but the exact role and route of transmission remains unknown. Approximately 35% of reported patients with MERS-CoV infection have died (Abdelrahman et al., 2020; Czeresnia et al., 2020; Di Mascio et al., 2020; Zhu et al., 2020).

SARS-CoV-2 is a novel severe acute respiratory syndrome coronavirus that causes variable symptoms including fever, cough, headache, fatigue, breathing difficulties, and loss of smell and taste (Czeresnia et al., 2020; Khalil et al., 2020; Matar et al., 2021). Symptoms usually begin within 14 days after exposure to the virus. The original source of viral transmission to humans remains unclear, but bats and pangolins have been implicated as animal reservoirs. At least a third of people who are infected remain asymptomatic. Most (81%) symptomatic patients develop mild to moderate symptoms, whilst 14% develop severe symptoms (dyspnea, hypoxia, or more than 50% lung involvement on imaging), and 5% suffer critical symptoms (respiratory failure, shock, or multiorgan dysfunction). Transmission is mainly *via* air contaminated by droplets and small airborne particles containing the virus (Abdelrahman et al., 2020; Czeresnia et al., 2020; Di Mascio et al., 2020; Zhu et al., 2020).

There are several variants of concern that have emerged. “Variants of Concern” have evidence of an increase in transmissibility, greater risk of severe disease, a significant reduction in neutralization by antibodies generated during previous infection or vaccination, or reduced effectiveness of treatments or vaccines (COVID-19 vaccines, 2022; Developing COVID-19 Vaccines, 2022). SARS-CoV-2 variants of interest are illustrated in Table 2.

**TABLE 2:** SARS-CoV-2 variants of concern (World Health Organization, 2020).

Name	First detected	Transmissibility	Disease severity
Omicron B.1.1.529	Botswana/South Africa November, 2021	Sublineages BA.1 and BA.1.1: Increased transmissibility compared with Delta	Sublineages BA.1 and BA.1.1: Decreased disease severity compared with Delta
		Sublineage BA.2: Increased transmissibility compared with BA.1	Sublineage BA.2: Similar disease severity as BA.1
Delta B.1.617.2	India Late, 2020	Increased transmissibility compared with Alpha	Increased disease severity compared with Alpha
Gamma P.1	Japan/Brazil January, 2021	Increased transmissibility compared with wild-type virus	Increased disease severity compared with wild-type virus
Beta B.1.351	South Africa July–August, 2020	Increased transmissibility compared with wild-type virus	Increased disease severity compared with wild-type virus
Alpha B.1.1.7	United Kingdom November, 2020	Increased transmissibility compared with wild-type virus	Increased disease severity compared with wild-type virus

## Pregnancy: A Period of Interest

Pregnancy is characterized by unique physiological changes to accommodate the growing fetus (Soma-Pillay et al., 2016). These physiological changes in the respiratory, cardiovascular, and immune systems predispose pregnant women to infections. The physiological adaptations of pregnancy are described in Table 3.

**TABLE 3:** Physiological changes of pregnancy that make pregnant women more susceptible to infection 2 (Soma-Pillay et al., 2016; Förger and Villiger, 2020).

Respiratory	• Increase in oxygen demand due to ∼15% increase in the metabolic rate and ∼20% increased consumption of oxygen. • 40–50% increase in minute ventilation due to an increase in tidal volume. • Arterial pO2 increases and arterial pCO2 decreases with a compensatory reduction in serum bicarbonate concentrations to 18–22 mmol/l. • Mild fully compensated respiratory alkalosis, arterial pH 7.4. • Decreased functional residual capacity. • Inspiratory reserve volume is reduced during early pregnancy, but increases in the third trimester, as a result of reduced functional residual capacity. • Subjective feeling of breathlessness without hypoxia.
Cardiovascular	• Cardiac output increases ∼20% by 8 weeks gestation mostly due to peripheral vasodilation which is mediated by endothelium-dependent factors. • A total of 25–30% fall in systemic vascular resistance in the first trimester, but increases to 40% by 20–28 weeks gestation. • Increase in stroke volume which is possibly due to the early increase in ventricular wall muscle mass and end-diastolic volume. • Heart is physiologically dilated, and myocardial contractility is increased. • Blood pressure decreases in the first and second trimesters but increases to pre-pregnancy levels in the third trimester. • Pulmonary vascular resistance (PVR) decreases significantly. • Serum colloid osmotic pressure is reduced by 10–15%. The colloid osmotic pressure/pulmonary capillary wedge pressure gradient is reduced by about 30%, making pregnant women particularly susceptible to pulmonary edema. • Changes may include a bounding or collapsing pulse and an ejection systolic murmur in ∼90% of pregnant women.
Immune	• A pro-inflammatory microenvironment which is crucial for normal implantation and parturition. • Monocyte activity in peripheral blood is higher during pregnancy than post-partum, and a tolerogenic subset of innate cells is at work in the placenta during pregnancy. • A downregulation of effector T cell activity and an expansion of regulatory T cells after delivery, the immunomodulatory effects mediated by fetal antigens and pregnancy hormones disappear, giving rise to T cell activity and persisting monocyte activity.

The physiological adaptations of pregnancy are a fine balance that occurs throughout the three trimesters. Any disequilibrium, such as that created by a viral infection, may lead to maternal and fetal complications (Wong et al., 2004; Soma-Pillay et al., 2016; Förger and Villiger, 2020; Novoa et al., 2021).

The severity of viral pneumonias in pregnant women as evidenced especially by influenza outbreaks, may be attributed to the shift from humoral to cell-mediated immunity. In the pregnant woman’s immune system innate immune cells like natural killer (NK) cells and monocytes respond more strongly to invading pathogens. However, this is associated with some negatively regulated adaptive immune responses (Förger and Villiger, 2020). The swollen upper respiratory tract and restricted lung expansion in the last trimester make the pregnant woman more susceptible to respiratory pathogens such as SARS-CoV-2. Whilst these physiological changes are essential to the development and survival of the maternal-fetal interface, it does render the pregnant woman relatively immunocompromised. In the pro-inflammatory stages, especially in the first trimester, a pregnant woman will be more likely to develop a cytokine storm, which in the presence of respiratory changes may make a pregnant woman less tolerant to the effects of hypoxia. Hypoxia may lead to vasoconstriction and ultimately intrauterine growth restriction. In the third trimester, the growing pregnancy enhances the physiological changes. A viral infection in this time period may result in an increased secretion of T-helper-2 (Th2) cytokines that suppress inflammation, thus preserving the anti-inflammatory environment (Wong et al., 2004; Czeresnia et al., 2020).

The emergence of SARS-CoV-2 led to reflection on the experiences of SARS-CoV-1 and MERS to guide management. The SARS-CoV-1 outbreak infected more than 8,000 people globally and resulted in 919 deaths. Twelve pregnant women were reported to have SARS-CoV-1, of which four (57%) of the seven women infected in the first trimester had miscarriages, four (80%) of the five women infected in the second and early third trimester had preterm births and two (40%) of the five women had intrauterine fetal growth restriction (Wong et al., 2004, 2020; Turan et al., 2020). Placental findings in SARS-CoV-1 affected pregnancies showed maternal thrombosis and avascular villi suggesting fetal thrombosis, and reduced placental weight which was proportional to interval from SARS-CoV-1 infection to delivery (Wong et al., 2004, 2021). Pregnant women with SARS-CoV-1 were more likely to require mechanical ventilation, renal replacement therapy for acute injury, and develop coronavirus-related seizures. There was no evidence of *trans-*placental spread of SARS-CoV-1 (Czeresnia et al., 2020), and there were no pediatric fatalities (Ng et al., 2004).

Of the 2,496 confirmed cases of MERS, 852 (34.1%) people died. In one study of 660 patients with MERS, 11 (1.7%) cases were diagnosed in pregnant women. Of these seven (64%) women were admitted to intensive care, there were three (27%) maternal deaths, and three (27%) stillbirths. Again, there was no evidence of vertical transmission of MERS (Alserehi et al., 2016; Czeresnia et al., 2020).

Whilst only a small proportion of pregnant women were infected with SARS-CoV-1 and MERS, these women suffered severe complications. No effective antiviral therapy or vaccine have been found for either of these coronavirus infections. This led us to anticipate severe adverse outcomes in pregnant women infected with SARS-CoV-2.

## Why Is COVID-19 Different?

The SARS-CoV-2 outbreak resulted in a pandemic with more than 3 million cases and approximately 230,000 deaths reported in the first 5 months, which is more deaths than cases in previous coronavirus outbreaks (see Table 1; Matar et al., 2021). Unlike SARS-CoV-1 and MERS-CoV, SARS-CoV-2 has reached epic global pandemic proportions with 520 million documented cases and 6,2 million deaths. Excess deaths have been estimated to be 14.9 million (range 13.3–16.6 million) and 18.2 million (Wang et al., 2021; World Health Organization, 2022b).

Pregnant women present with similar symptoms, though less frequent than the non-pregnant population. The majority of pregnant women with SARS-CoV-2 are asymptomatic and are usually screened due to more frequent contact with healthcare services and pregnancy-related admissions such as for delivery. Pregnant women with SARS-CoV-2 present with obstetric complications (Table 4) such as preterm labor, pre-eclampsia or HELLP (hemolytic anemia, elevated liver enzymes, low platelets)-syndrome like illnesses. Preterm births are increased in women infected with SARS-CoV-2, but are iatrogenic rather than a direct consequence of viral infection (Khalil et al., 2020; Novoa et al., 2021). Pregnant women with a body mass index (BMI) more than 35 kg/m^2^, pre-eclampsia, or underlying cardiopulmonary conditions should receive greater care when infected. In addition, women with confirmed SARS-CoV-2 infection should be monitored closely post-partum due to the risk of deterioration (Czeresnia et al., 2020; Khalil et al., 2020; Sakowicz et al., 2020; Novoa et al., 2021).

**TABLE 4:** Pregnancy related complications of SARS-CoV-2 (Goldshtein et al., 2021, 2022; Shimabukuro et al., 2021; Morgan et al., 2022).

	SARS-CoV-2 findings	
**General symptoms** COVID-19 overlap with symptoms of normal pregnancy*	Pregnant people are more likely to be asymptomatic Common symptoms: • Cough • Fever • Myalgias • Headache • Dyspnoea* • Sore throat • Diarrhoea • Nausea/vomiting* • Anosmia or other smell abnormalities • Ageusia or other taste abnormalities • Rhinorrhoea and/or nasal congestion* • Chills/rigors • Fatigue*	Pregnancy can worsen the clinical course of SARS-CoV-2, especially in women of older age (especially >35 years), obesity, preexisting medical comorbidities (particularly hypertension, diabetes, or >1 comorbidity), and being unvaccinated. Consider other viral and bacterial respiratory infections depending on local prevalence.
	Laboratory findings: • Thrombocytopenia • Elevated liver enzymes • Autoimmune hemolysis • Prolonged prothrombin time • Elevated D-dimer • Elevated procalcitonin • Elevated C-reactive protein (CRP) levels • Positive lupus anticoagulant screen • Low fibrinogen levels • Acute kidney injury	
**Overlap with pregnancy specific conditions**	Thrombocytopenia elevated liver enzymes	Consider preeclampsia, HELLP (hemolysis, elevated liver enzymes, low platelets) syndrome. Symptoms also overlap: Headache, acute cerebrovascular disease, and seizures. Hypertension is a feature of preeclampsia but not usually of SARS-CoV-2.
	Acute kidney injury	Can also be a severe complication of obstetric disorders, such as pre-eclampsia with severe features, abruptio placentae, or hemorrhagic shock. Uterine bleeding is a prominent feature of the last two disorders but not for SARS- CoV-2 or preeclampsia.
**Effects on pregnancy**	No effect on miscarriage risk • No effect on risk of congenital anomalies • Increased in severe disease: ° Preterm birth ° Caesarean section ° Stillbirths • Increased risk of pre-eclampsia and HELLP syndrome	
**Vertical transmission**	Extent of vertical transmission (in utero, intrapartum, early postnatal period) remains unclear, but may occur *via* hematogenous or ascending route.	

The symbol * indicates symptoms that overlap with normal pregnancy changes.

In light of its increased transmissibility and spread especially with the Omicron variant, SARS-CoV-2 has resulted in a greater global burden of disease in pregnancy than SARS-CoV-1 and MERS (Zhu et al., 2020).

Pregnant women were amongst the highest risk cohorts in both the SARS-CoV-1 and MERS outbreaks. which is not the case with SARS-CoV-2 (Abdelrahman et al., 2020; Khalil et al., 2020; Zhu et al., 2020; Matar et al., 2021). Contrary to expectations, the pregnancy associated morbidity associated with SARS-CoV-2 is less severe than SARS-CoV-1 and MERS (Sakowicz et al., 2020). Compared with intensive care unit (ICU) admission and mortality rates in the previous coronavirus outbreaks, only 10% of pregnant women infected with SARS-CoV-2 required ICU admission for respiratory support (Novoa et al., 2021). The underlying reason for this still remains unclear but several theories have been postulated.

1) There may be a protective mechanism/barrier within the placenta which relies on the presence or absence of certain receptors/pathways (Sadeghi et al., 2021).
2) COVID-19 shows some peculiar pathogenetic, epidemiological, and clinical features which to date are not completely understood (Petrosillo et al., 2020). Some studies reveal that SARS-CoV-2 is very similar in structure and pathogenicity with SARS-CoV-1, but the most important structural protein, i.e., the spike protein (S), is slightly different in these viruses (Rabaan et al., 2020) and undergoing significant mutations over time leading to changing transmissibility and possibly severity of disease (Abdullah et al., 2022).
3) The familial hemophagocytic lymphohistiocytosis (HLH) gene mutations, which was found that patients with H1N1 influenza who experienced the “cytokine storm,” has not been found in COVID-19. Furthermore, the widespread infiltration of monocytes/macrophages in the lung tissue of COVID-19 patients and the peripheral monocyte trafficking and subsequent differentiation into macrophages in the lungs of COVID-19 patients contributes to pro- inflammatory responses and further activation of innate immune cells. The changes in the innate immune system during pregnancy, also, involve the pattern recognition receptors Toll-like receptors (TLRs). Infection with COVID-19 leads to pyroptosis of host cells and the release of danger associated molecular patterns (DAMPs) that can act as ligands for TLR molecules and trigger a greater inflammatory response (Sadeghi et al., 2021).

Further studies are needed to determine which factors result in higher susceptibility or are protective against COVID-19 during pregnancy.

Another question that has arisen during the SARS-CoV-2 pandemic is “Does SARS-CoV-2 cause pre-eclampsia?” Whilst there is an apparent link, probably due to the placental affectation by the viral infection, it is not clear whether this association is causal (Gajbhiye et al., 2020; Wong et al., 2021; Khalil et al., 2022). The risk factors for pre-eclampsia and SARS-CoV-2 infection, namely, Black or Asian ethnicity, overweight/obesity, and underlying chronic conditions overlap, which may be confounding factors. Whilst there are plausible theories that SARS-CoV-2 causes pre-eclampsia, clear evidence is lacking (Khalil et al., 2022).

Reports on vertical transmission of SARS-CoV-2 are conflicting. Three (9.1%) neonates born from 33 COVID-19 infected women in China were thought to have contracted SARS-CoV-2 *in utero* (Sakowicz et al., 2020). In a review of 441 cases Gajbhiye et al. (2020) report a vertical transmission rate of approximately 8%. Whilst the presence of SARS-CoV-2 in the bloodstream is evident, and that it uses the membrane bound ACE2 receptor, which is expressed in human placenta, to gain access to its target cell, current data do not support the premise of transmission *in utero*, even though the virus has been isolated in placenta in severe cases. This may be due to the placenta’s immunological barrier which prevents entry of pathogens (Wong et al., 2021). Current evidence does not support transmission *via* breastfeeding (Novoa et al., 2021).

SARS-CoV-2 unlike previous outbreaks, has been associated with an increase in gender-based violence and an exacerbation of mental health disorders. Pregnancy involves several changes at the social, biological, and psychological levels in mothers and their families. Pregnant and post-partum women have high prevalence rates of anxiety, depression, and insomnia, but during disasters, such as the current pandemic, the prevalence of mental disorders in prenatal and postnatal women are significantly higher than those in the general population. This may be attributed to the quarantine measures and disruptions in medical practices (Yan et al., 2020). Interestingly, a recent study reported that lockdown restrictions increase gestational diabetes mellitus (GDM), probably due to stress.

Over the last few months we have seen the emergence of the Omicron variant which spreads more easily than the original virus that causes COVID-19 and the Delta variant (Centers for Disease Control and Prevention, 2022). Infection with the Omicron variant causes less severe disease than infection with prior variants, although some people may still have severe disease, need hospitalization, and could die from the infection with this variant (Centers for Disease Control and Prevention, 2022). Unvaccinated individuals who contract the Omicron variant are at greatest risk of severe disease and adverse outcomes (Engjom et al., 2022).

SARS-CoV-2 in pregnancy presents with many challenges on the women’s physiological, immunological, and social level, it further challenges the health system [service provision, essential service continuation, healthcare provider safety, personal protective equipment (PPE), and labor/delivery practices, etc.] particularly in developing countries. PPE to protect against the spread of COVID-19 include wearing a medical mask, gown, glove, and eye protection. The use of PPE by attending healthcare workers during labor and delivery remains controversial. N95 (non-oil respirator) respirators, which are respirators that offer a higher level of protection instead of a facemask, are recommended for those healthcare workers performing or present during an aerosol-generating procedures (AGP). Despite the second stage of labor lasting up to 4 h and the healthcare worker being in close contact to women who are exerting extreme effort and may exhale vigorously, cough, shout, and vomit during this time, the second stage of labor is not considered an AGP. However, it is recommended that labor and delivery personnel exercise caution and protect themselves and other patients. The implications, indirect effects, and evidence is still evolving, and more research and data is needed (Adam et al., 2020; Palatnik and McIntosh, 2020).

## Vaccines and Pregnancy

The SARS-CoV-2 pandemic has showcased the rapid advances in science and technology with the speedy development of vaccines (COVID-19 vaccines, 2022; Developing COVID-19 Vaccines, 2022). The development of safe effective vaccines provides an ideal opportunity to alleviate some of burdens facing maternal and infant health. Vaccines are highly effective at preventing morbidity and mortality. It is recommended that all unvaccinated women planning pregnancy or pregnant and breastfeeding women receive the COVID-19 vaccine and booster doses as per local protocols, as soon as possible. The COVID-19 vaccines may be administered at the same time as other vaccines routinely administered in pregnancy or at the same time as Anti-D immunoglobin.

None of the recommended COVID-19 vaccines [Pfizer/BioNTech (Comirnaty) by Fosun Pharma, Pfizer; Moderna (Spikevax); Janssen/Johnson & Johnson by Janssen Pharmaceutical Companies] contain virus that replicates. Thus, they do not cause disease, but non-specific side-effects from activation of the immune system may occur (Brinkley et al., 2022). Women can be assured that data supports the safety of vaccine in pregnancy and lactation. Benefits of vaccination include reductions in maternal SARS-CoV-2 infection, severity of maternal COVID-19, perinatal death, COVID-19-related hospitalization among infants up to 6 months of age (American College of Obstetricians and Gynecologists, 2022; Brinkley et al., 2022; Fu et al., 2022). No harmful effects on fertility, embryo/fetal development, pregnancy outcome, parturition, or short-term postnatal development of offspring has been reported in association with vaccines. Side effects are similar in pregnant and non-pregnant females (Brinkley et al., 2022).

Whilst vaccine hesitancy initially appeared to be lower in lower- and middle-income countries (LMICs), that trend is changing as vaccines become more accessible. In South Africa, only 41,73% of women aged 18–34 have been vaccinated making the lack of vaccination the single biggest risk factor for severe disease and death in pregnant women (Latest Vaccine Statistics, 2022).

The inequitable access to vaccines by pregnant women in the global south compared to their peers in rich countries has significantly skewed the burden of severe disease and death across the geographic divide and requires ongoing urgent attention. The education of pregnant women and communities in these areas and access to safe SARS-CoV-2 vaccines will further the endeavors of equity in healthcare in the world’s most deserving areas (Kalafat et al., 2021; Magee et al., 2021; Vale et al., 2021).

## Health System Strengthening

The COVID-19 pandemic has highlighted the need for health system strengthening particularly in LMICs. Early in the pandemic, Robertson et al. (2020) warned of an 8–38% increase in maternal mortality in LMICs if routine and emergency services were not maintained by health systems. The global response to the COVID-19 pandemic has largely been centered on lockdowns to ensure physical distancing whilst trying to maintain essential health services. For many countries, this approach has resulted in a shift from essential healthcare services to providing emergency services together with COVID-19 care (Ahmed et al., 2021).

Lower- and middle-income countries, like South Africa, observed a 30% increase in maternal mortality during the first wave of COVID-19 infections (Pattinson et al., 2020). This increase in maternal deaths coincided with a significant decrease in usage of reproductive health services including contraceptive usage and attendance at termination of pregnancy clinics. Additionally, the lockdown resulted in a movement of women from metropolitan cities (areas of work) to their homes in rural areas. Rural provinces of Mpumalanga and Limpopo experienced excess pressure on already under-resourced health systems while metropolitan cities were overburdened with severe COVID-19 infection and an inability to manage other routine emergencies (Pattinson et al., 2020).

Important components of COVID-19 care include the provision of screening facilities, availability of PPE and vaccine coverage for healthcare workers. Ahmed et al. (2021) reported a shortage of screening facilities and PPE in three LMICs. There has been global disparities in vaccine distribution with high-income countries purchasing excess vaccines for their citizens with low vaccine coverage in most African countries. The World Health Organization has reported that only 1 in 4 (27%) African health workers are fully vaccinated against COVID-19 (World Health Organization, 2022a). This means that African healthcare workers remain dangerously exposed to severe COVID-19 infection. High vaccine coverage amongst healthcare workers is essential not only for their own protection but also for their patients and to ensure that health systems remain operation during a time of extreme need.

## The Challenges of the 21st Century

The SARS-CoV-2 pandemic has showcased the rapid advances in science and technology with the speedy development of vaccines (COVID-19 vaccines, 2022; Developing COVID-19 Vaccines, 2022). However, our achievements and unprecedented advances have posed their own challenges. We have seen that globalization and ease of travel have facilitated the spread of SARS-CoV-2. Advances in speed of communication and access to information (and misinformation) have allowed vaccine hesitancy and conspiracy theories to thrive. In addition, the rapid emergence of new strains of SARS-CoV-2 has resulted in political antagonism, especially directed at low-middle income countries.

Despite all our advances, the 21st century has also unmasked the prevailing social injustices, especially in healthcare. Pregnant women and children in under-resourced settings bear the brunt of morbidity and mortality, especially related to basic human needs such as access to food, safe drinking water and basic healthcare. This is especially true with the SARS-CoV-2 pandemic.

## Author Contributions

All authors have contributed equally to the conception and development of the manuscript, contributed to the article, and approved the submitted version.

## Conflict of Interest

The authors declare that the research was conducted in the absence of any commercial or financial relationships that could be construed as a potential conflict of interest.

## Publisher’s Note

All claims expressed in this article are solely those of the authors and do not necessarily represent those of their affiliated organizations, or those of the publisher, the editors and the reviewers. Any product that may be evaluated in this article, or claim that may be made by its manufacturer, is not guaranteed or endorsed by the publisher.
